# Outcomes of elderly patients with relapsed refractory multiple myeloma (RRMM) treated with teclistamab: a multicenter study from the U.S. Multiple Myeloma Immunotherapy Consortium

**DOI:** 10.1038/s41408-025-01297-7

**Published:** 2025-05-09

**Authors:** Oren Pasvolsky, Danai Dima, Lei Feng, Wenli Dong, Tiffany Richards, James A. Davis, Aimaz Afrough, Mariola Vazquez-Martinez, Aishwarya Sannareddy, Utkarsh Goel, Rahul Banerjee, Jack Khouri, Frances Cervoni, Mahmoud R. Gaballa, Alex Lieberman-Cribbin, Masooma Shifa Rana, Kelley Julian, Christopher J. Ferreri, Leyla Shune, Shaun DeJarnette, Evguenia Bhurtel, Sandra Susanibar Adaniya, Andrew Portuguese, Hitomi Hosoya, Lekha Mikkilineni, Gurbakhash Kaur, Adriana Rossi, Megan M. Herr, Daniel Schrum, Chenyu Lin, Shahzad Raza, Yi Lin, Shonali Midha, Nadeem Omar, Shebli Atarsh, Joseph McGuirk, Douglas Sborov, Peter Voorhees, Faiz Anwer, Melissa Alsina, Ciara Freeman, Alfred L. Garfall, Beatrice M. Razzo, Surbhi Sidana, Andrew J. Cowan, Larry D. Anderson Jr, Doris K. Hansen, Shambavi Richard, Krina K. Patel, Hans C. Lee, Ariel Grajales-Cruz

**Affiliations:** 1 MD Anderson Cancer Center, Houston, TX USA; 2 Fred Hutchinson Cancer Center, Seattle, WA USA; 3 Cleveland Clinic Lerner College of Medicine, Case Western Reserve University School of Medicine, Cleveland, OH USA; 4 Medical University of South Carolina, Charleston, SC USA; 5 Myeloma, Waldenstrom’s, and Amyloidosis Program, Simmons Comprehensive Cancer Center, UT Southwestern Medical Center, Dallas, TX USA; 6 Moffitt Cancer Center, Tampa, FL USA; 7 Mount Sinai School of Medicine, New York, NY USA; 8 Stanford University, Palo Alto, CA USA; 9 Huntsman Cancer Institute, University of Utah, Salt Lake City, UT USA; 10 Atrium Health Levine Cancer Institute, Wake Forest University School of Medicine, Charlotte, NC USA; 11 University of Kansas Medical Center, Kansas City, KS USA; 12 Abramson Cancer Center and Perelman School of Medicine, University of Pennsylvania, Philadelphia, PA USA; 13 Roswell Park Comprehensive Cancer Center, Buffalo, NY USA; 14 Duke University Cancer Institute, Durham, NC USA; 15 Division of Hematology, Mayo Clinic, Rochester, MN USA; 16 Dana-Farber Cancer Institute, Boston, MA USA

**Keywords:** Myeloma, Epidemiology

## Abstract

Teclistamab, a BCMA-directed bispecific antibody, received regulatory approval for relapsed/refractory multiple myeloma (RRMM) based on the MajesTEC-1 study. Despite the fact that myeloma is primarily a cancer of elderly adults, only 15% of MajesTEC-1 participants (*n* = 24) were ≥75 years old. In this multicenter retrospective study, we report real-world outcomes of a large cohort of older RRMM patients treated with teclistamab. Of 385 analyzed patients, 83 (22%) were in the older group (age ≥75) and 302 (78%) in the younger group (age <75). Compared to the younger group, the older group had less adverse baseline disease characteristics, including a lower incidence of high-risk cytogenetics (44.6% vs. 57.9%, *p* = 0.03) and extramedullary disease (22% vs. 40%, *p* = 0.02). There were no significant differences in rates of any-grade CRS (52% vs. 59%, *p* = 0.27), any-grade ICANS (19% vs. 13%, *p* = 0.12), and overall response rate (62% vs. 53%, *p* = 0.17) between the older and younger groups. In multivariable analysis, age was not significantly associated with survival outcomes. Our findings suggest that teclistamab is safe and efficacious in well-selected patients ≥75 years old, and advanced age alone should not preclude teclistamab administration.

## Background

Teclistamab (Tec) is the first-in-class bispecific T-cell antibody approved for the treatment of patients with relapsed/refractory multiple myeloma (RRMM) [1]. This approval was based on the results of the pivotal phase I-II MajesTEC-1 trial, which evaluated Tec, a dual targeting antibody that binds both CD3 and B-cell maturation antigen (BCMA), in 165 patients with RRMM after at least three prior lines of therapy [2]. The overall response rate (ORR) was 63%, and the median progression-free survival (PFS) was 11.3 months. Median overall survival (OS), as reported after two years of follow-up, was 21.9 months [3]. The overall incidence of cytokine release syndrome (CRS) was 72.1% (*n* = 119, 0.6% grade 3/4) and of neurotoxicity 14.5% (*n* = 24, 0.6% grade 3/4).

The median age at diagnosis of MM is 69 years, and approximately one-third of patients are diagnosed at the age of 75 years or above [1]. Nonetheless, the elderly are underrepresented in clinical trials for MM. In the MajesTEC-1 study, only 15% of patients (*n* = 24) were aged ≥75 years at enrollment [2]. While underpowered to draw any definitive conclusions, the ORR was numerically lower in patients aged ≥75 (54%) than in younger patients (65%). In an analysis of 177 MM clinical trials registered in ClinicalTrials.gov between 2000 and 2016, 32% of enrolled participants were older than 65 years [4]. There has also been a downward trend in enrolling older patients over time, with only 23% of patients enrolled in clinical trials in 2016 being aged >65 years. Furthermore, despite the continuous development of novel drugs for patients with MM [5], the elderly have not experienced the same survival benefit as younger patients [6].

The addition of T-cell redirecting immunotherapy modalities, such as Tec, to the armamentarium against MM has put forth an opportunity and a challenge of incorporating these therapies in the management of older myeloma patients. Increased comorbidity burden and reduced organ reserves in this subgroup could have potential effects on the safety profile of Tec, especially in regards to CRS and ICANS [7].

There are limited data to date characterizing the safety and efficacy of Tec in older patients with MM [8, 9] to inform the feasibility of Tec administration in this patient population. Therefore, we conducted a multicenter retrospective study on behalf of the United States Multiple Myeloma Immunotherapy Consortium to evaluate the real-world safety and efficacy of a large cohort of elderly (≥75 years) RRMM patients treated with Tec.

## Methods

### Patient and data selection

We conducted a retrospective multicenter study of all consecutive patients with MM across 13 United States academic medical centers who participated in the United States Multiple Myeloma Immunotherapy Consortium. At each participating center, an Institutional Review Board approval for retrospective studies through the Consortium was obtained, including waiver of informed consent. Tec was administered according to the standard of care practice used at each center, including step-up dosing protocols, supportive care, and timing of response assessments. A secure REDCap interface [10] was used to collect relevant datapoints, with a margin of ±7 days for each data point. High-risk cytogenetic abnormalities (HRCA) were defined as deletion (del) 17p, translocation (t) (4;14), *t*(14;16), and/or gain or amplification of 1q21. Extramedullary disease (EMD) included both para-skeletal plasmacytomas and extramedullary soft tissue plasmacytomas [11]. Cytokine release syndrome (CRS) and immune effector cell-associated neurotoxicity syndrome (ICANS) assessment was based on the American Society for Transplantation and Cellular Therapy consensus grading [12]. International Myeloma Working Group (IMWG) consensus criteria were used to assess response to Tec [13]. The follow-up data cutoff was 30 April 2024.

### Statistical analyses

The cohort was divided into two age groups, patients aged ≥75 years (≥75 group) and those aged <75 years (<75 group), with the age cutoff of 75 chosen based on its previous use in the MajesTEC-1 trial of Tec. Descriptive statistics were used to summarize patient demographics, disease characteristics, and clinical outcomes. The cutoff values to dichotomize the continuous variables, such as C-reactive protein and ferritin, were the upper quartile for each variable. The association between two categorical variables was evaluated using the Chi-square test or Fisher’s exact test. Differences between patient groups in a continuous variable were evaluated using the Wilcoxon rank sum test. A multivariable logistic regression model was fitted to estimate the effects of important variables on best overall response and survival outcomes. For time-to-event analyses, including PFS and OS, the Kaplan–Meier method was used, and the median time to event was calculated in months with a 95% confidence interval. Due to a significant imbalance of baseline patient characteristics in the two age cohorts, direct statistical comparisons in time-to-event end-points between the two age groups were not performed. Cox proportional hazards models were used for multivariable analysis. Schoenfeld residual was used to check the proportional hazards assumption. The baseline variables that had a *p*-value less than 0.05 for PFS or OS from the univariate analysis and without significant missing information were included in the full models. A backward selection method was used, and the final model included the covariates with a *p*-value less than 0.05.

Statistical software SAS 9.4 (SAS, Cary, NC) and S-Plus 8.2 (TIBCO Software Inc., Palo Alto, CA) were used for all the analyses.

## Results

### Patient characteristics

Three hundred eighty-five patients were included in the analysis, and baseline characteristics are summarized in Table 1. A total of 83 (22%) were in the ≥75 age group (median age 78, range 75–92) and 302 (78%) in the <75 group (median age 65, range 31–74). Compared to patients in the <75 group, those in the ≥75 group were more often white (78% vs. 63%, *p* = 0.009). Both the ≥75 and <75 age groups had a median of 6 prior lines of therapy, and a similar proportion of patients had a performance status of Eastern Cooperative Oncology Group (ECOG) ≥ 2 at the start of Tec (29% vs. 24%, respectively; *p* = 0.37). Both age groups had a high proportion of patients with triple-class-refractory disease (77% vs. 85%, *p* = 0.06) and patients who would have been ineligible for the MajesTEC-1 study (73% vs. 80%, *p* = 0.20). Patients in the ≥75 group had a lower incidence of some adverse MM risk features compared to the <75 group, including HRCA (44.6% vs. 57.9%, *p* = 0.03), ultra high-risk (≥2 HRCA) MM (12% vs. 24%, *p* = 0.02), EMD (22% vs. 40%, *p* = 0.02), hemoglobin <8 g/dL (10% vs. 21%, *p* = 0.02) and albumin <3 g/dL (6% vs. 19%, *p* = 0.04) at the time of starting Tec. Moreover, a lower number of pts in the ≥75 group had prior autologous stem cell transplantation (43% vs. 72%, *p* < 0.0001) or prior BCMA-directed therapy (33% vs. 55%, *p* = 0.0003). The median time from diagnosis of MM to start of Tec was longer for patients in the ≥75 group (7.21 years vs. 5.81 years, *p* = 0.02).

**Table 1:** Patient characteristics.

Measure	Age group		*p*-value
	≥75 (*N* = 83)	<75 (*N* = 302)	
**Age, median (range)**	78 (75–92)	65 (31–74)	
**Gender**, ***n*** **(%)**			0.35
Male	40 (48%)	163 (54%)	
Female	43 (52%)	139 (46%)	
**Race**, ***n*** **(%)**			0.0086
White	65 (78%)	190 (63%)	
Non-White	18 (22%)	112 (37%)	
**ECOG** ≥ **2**^**a**^, ***n*** **(%)**			0.37
Yes	23 (29%)	65 (24%)	
No	57 (71%)	208 (76%)	
NA	3	29	
**MajesTEC-1 eligible**, ***n*** **(%)**			0.20
Yes	22 (27%)	61 (20%)	
No	61 (73%)	241 (80%)	
**Baseline CrCl** < **30** **ml/min**, ***n*** **(%)**			0.59
Yes	11 (14%)	34 (11%)	
No	70 (86%)	265 (89%)	
NA	2	3	
**Baseline LDH** > **ULN**, ***n*** **(%)**			0.58
Yes	34 (43%)	120 (46%)	
No	46 (58%)	141 (54%)	
NA	3	41	
**Cytogenetic risk**, ***n*** **(%)**			0.0301
High^b^	37 (45%)	175 (58%)	
Standard	46 (55%)	127 (42%)	
**Ultra high-risk myeloma**^**c**^, ***n*** **(%)**			0.0233
Yes	10 (12%)	71 (24%)	
No	73 (88%)	231 (77%)	
**Bone marrow PC** > **50%**, ***n*** **(%)**			0.23
Yes	5 (21%)	43 (36%)	
No	19 (79%)	78 (64%)	
NA	59	181	
**EMD at baseline**, ***n*** **(%)**			0.0018
Yes	18 (22%)	121 (40%)	
No	65 (78%)	181 (60%)	
**Prior lines of therapy** ≥ **6**, ***n*** **(%)**			0.22
Yes	43 (52%)	175 (59%)	
No	40 (48%)	127 (42%)	
**Triple-class refractory**^**d**^, ***n*** **(%)**			0.06
Yes	63 (77%)	257 (85%)	
No	20 (24%)	45 (15%)	
**Penta-class refractory**^**e**^, ***n*** **(%)**			0.15
Yes	24 (30%)	118 (39%)	
No	59 (71%)	184 (61%)	
**Prior autologous transplant**, ***n*** **(%)**			<0.0001
Yes	35(43%)	218 (72%)	
No	47 (57%)	84 (28%)	
**Prior anti-BCMA agent**, ***n*** **(%)**			0.0003
Yes	27 (33%)	166 (55%)	
No	56 (68%)	136 (45%)	
**Prior anti-GPRC5D agent**, ***n*** **(%)**			1.00
Yes	2 (2.4%)	9 (3%)	
No	81 (98%)	293 (97%)	

*BCMA* B-cell maturation antigen, *CrCl* Creatinine clearance, *ECOG* Eastern Cooperative Oncology Group, *EMD* extramedullary disease, *GPRC5D* G protein-coupled receptor class C group 5 member D, *LDH* lactate dehydrogenase, *NA* not available, *PC* plasma cells, *ULN* upper limit of normal.

^a^At the start of teclistamab.

^b^Defined as del(17p), *t*(4;14), *t*(14;16) and/or gain or amplification of 1q21.

^c^Defined as having ≥2 high-risk cytogenetic abnormalities.

^d^Defined as being refractory to a proteasome inhibitor, an immunomodulatory agent, and an anti-CD38 monoclonal antibody.

^e^Defined as being refractory to lenalidomide, pomalidomide, bortezomib, carfilzomib, and an anti-CD38 monoclonal antibody.

### Safety

**Fig. 1:**
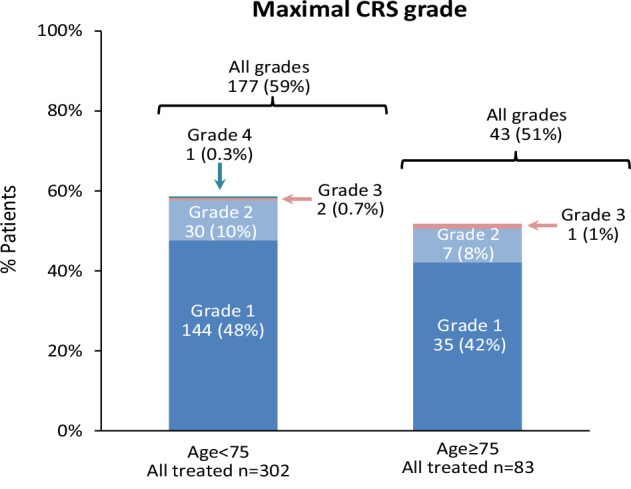
Maximal CRS grade according to age group.

An accelerated step-up dosing schedule (defined as having at least one of the step-up dose intervals of less than three days apart) was used in 62% of the ≥75 group and 58% of the <75 group (*p* = 0.56). There were 12 patients (14%) in the ≥75 group and 33 (11%) in the <75 group (*p* = 0.35) who received outpatient step-up dosing. There was no significant difference in rates of any-grade CRS (52% vs. 59%, *p* = 0.27) or grade 2–4 CRS (10% vs. 11%, *p* = 0.74) between the ≥75 and <75 age groups, respectively. Four patients in the entire cohort developed CRS grade 3–4, one of whom was in the ≥75 group that developed grade 3 CRS (Fig. 1). Median time from start of Tec to onset of CRS and median time to maximal CRS were both 3 days, without a significant difference between the two groups (*p* = 0.60 and *p* = 0.62, respectively). Twenty-six patients (31%) in the older age group received tocilizumab for CRS, compared to 121 (40%) in the younger age group (*p* = 0.54). Fourteen patients (17%) in the older age group received steroids for CRS, compared to 53 (18%) in the younger age group.

**Fig. 2:**
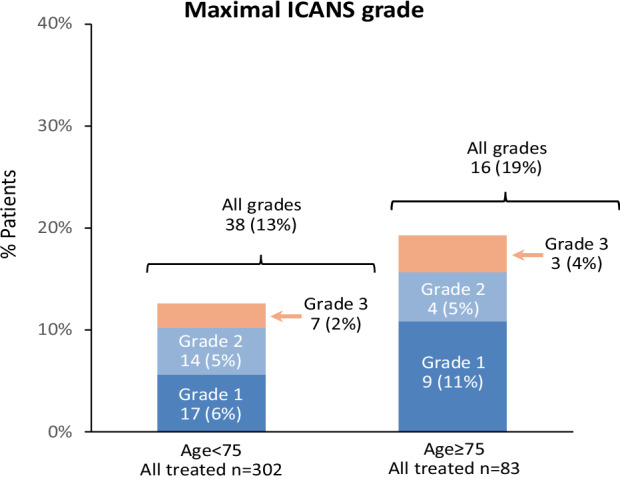
Maximal ICANS grade according to age group.

There was no significant difference between the two age groups in the rate of any-grade ICANS: 19% in the ≥75 group and 13% in the <75 group, *p* = 0.12. Ten patients developed grade 3 ICANS (none grade 4), three of whom were in the ≥75 group (Fig. 2). Median time from start of Tec to onset of ICANS was numerically shorter in the ≥75 group (3 days, range 0–25 days) compared to the <75 group (4 days, range 0–21 days), though not reaching a statistically significant difference (*p* = 0.89). Likewise, median time to maximal ICANS was numerically shorter in the ≥75 group (4 days, range 0–25 days) compared to the <75 group (5 days, range 0–22 days, *p* = 0.23). Moreover, the rate of a composite outcome of CRS grade 2–4 and/or any-grade ICANS was similar in both age groups (23% in the ≥75 group, and 21% in the <75 group).

A total of 20 patients in the ≥75 group and 118 patients in the <75 group died during follow-up. The most common cause of death in both age groups was progression of MM (70% of the total deaths in the ≥75 group and 75% in the <75 group), followed by infections (25% of the total deaths and 10%, respectively). The leading causes of infections leading to death in both age groups were sepsis and pneumonia (Supplementary Table 1). There was a trend toward lower rates of early death in the ≥75 group vs. the <75 group, when evaluating rates of death at 3 months (15% vs. 25%, respectively; *p* = 0.05) and at 6-months (22% vs. 31%, *p* = 0.1) after start of Tec.

### Efficacy

**Fig. 3:**
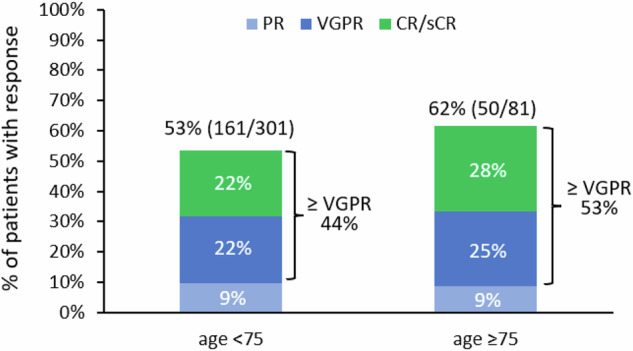
Overall response according to age group.

There was no significant difference in ORR between the ≥75 group and the <75 group (62% vs. 53%, *p* = 0.17) and ≥very good partial response (VGPR) rate (53% vs. 44%, *p* = 0.14) (Fig. 3).

**Fig. 4:**
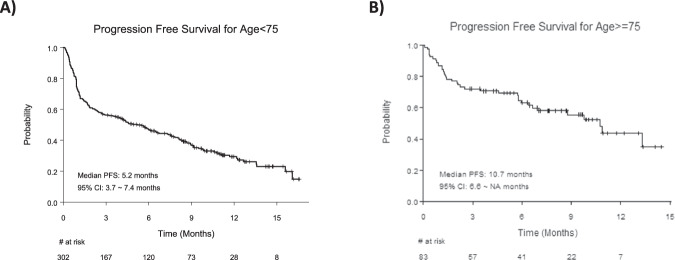
Kaplan–Meier progression-free survival curves. **A** Progression free survival in the <75 years age group. **B** Progression free survival in the ≥75 years age group.

The median follow-up period was 8.7 months (95% CI 7.6–9.9 months) in the ≥75 group and 10.4 months (95% CI 9.9–11.2 months) in the <75 group. The unadjusted median PFS in the ≥75 group was 10.7 months (95% CI 6.6–NR) and was 5.2 months (95% CI 6.6–NR) in the <75 group (Fig. 4).

**Fig. 5:**
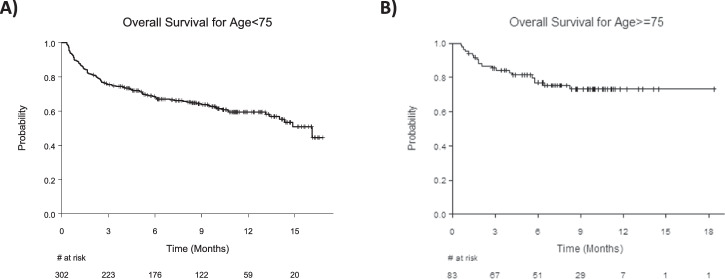
Kaplan–Meier overall survival curves. **A** Overall survival in the <75 years age group. **B** Overall survival in the ≥75 years age group.

At the time of data cutoff, the median overall survival (OS) for patients in the ≥75 group had not been reached (95% CI NR–NR) and was 16.1 months (95% CI 14.0–NR) in the <75 group (Fig. 5). The OS rate at 12 months was 73% (95% CI 63%–84%) in the ≥75 group and 59% (95% CI 54%–66%) in the <75 group.

In multivariable analysis for the entire cohort, age ≥75 was not significantly associated with either PFS (HR 1.15, 95% CI 0.72–1.84; *p* = 0.55) or OS (HR 1.67, 95% CI 0.84–3.33; *p* = 0.15).

Factors that were associated with significantly worse PFS in multivariable analysis in the entire cohort included prior anti-BCMA targeting agent (HR 1.60, *p* = 0.0090), baseline ferritin ≥ upper quartile (HR 2.00, *p* = 0.0010), baseline CRP ≥ upper quartile (HR 1.61, *p* = 0.0146), baseline Hb <8 (HR 1.90, *p* = 0.0032) and presence of either plasma cell leukemia (HR 4.93, *p* = 0.0128) or EMD (HR 1.67, *p* = 0.0048) prior to start of Tec. Full details of multivariable analysis for PFS are presented in Supplementary Table 2.

Factors that were associated with significantly worse OS in multivariable analysis in the entire cohort included ECOG ≥ 2 (HR 1.92, *p* = 0.0101), baseline CRP ≥ upper quartile (HR 2.58, *p* = 0.0002), baseline platelets <50 × 10^9^/L (HR 2.54, *p* = 0.0005), baseline absolute lymphocyte count <0.5 × 10^9^/L (HR 1.97, *p* = 0.0062) and presence of plasma cell leukemia prior to start of Tec (HR 5.24, *p* = 0.0320). Full details of multivariable analysis for OS are presented in Supplementary Table 3.

## Discussion

We report a large multicenter study showing favorable outcomes for elderly RRMM patients ≥75 years of age treated with Tec in the real-world setting. We observed similar rates of overall and high-grade CRS or ICANS between the two age groups. The ORR was 62% in the ≥75 age group and 53% in the <75 group. The older group had an unadjusted median PFS of 10.7 months with a median OS not reached. The younger group had an unadjusted median PFS of 5.2 months and median OS of 16.1 months. Despite numerical differences in PFS and OS, age was not associated with survival outcomes in the multivariable analysis between the two age groups, likely reflecting patient selection and prescribing patterns in the early era of access to commercial anti-BCMA bispecific therapy with Tec, with more stringent selection patients in the older cohort who were fit with favorable disease characteristics.

The global population is gradually aging, and a report by the United Nations predicts that the number of people over the age of 80 will more than double its current number by the year 2050 [14]. The incidence of MM, a disease of the elderly, is also expected to increase almost twofold over the next quarter of a century [15], with MM patients living longer and requiring safe and efficacious treatment options. In this context, it is important that new anti-myeloma agents are evaluated in the older patient population. Previous research has shown that autologous stem cell transplantation can result in excellent outcomes in selected elderly MM patients aged ≥75 years [16]. A recent real-world study evaluated the safety and efficacy of idecabtagene vicleucel in older MM patients (aged ≥65 years) [17]. The ORR was 86.7% and 84.0% in the ≥65 group and <65 group, respectively (*p* = 0.92). There was numerically longer median PFS in the older cohort (9.1 months vs. 7.4 months), not reaching statistical significance (*p* = 0.39). The 12-month OS rates were 68% and 76.5% in the older and younger age groups, respectively. Coupled with the current report, it is encouraging that the emerging T-cell redirecting therapies are showing good tolerability and efficacy in older real-world cohorts. Moving forward, it is imperative that new treatment modalities be prospectively evaluated in a manner that reflects the real-world age distribution, with an emphasis on reducing barriers for elderly patients to enroll in clinical trials.

Response rates and survival outcomes in the elderly cohort of the current study were similar to the entire cohort reported on the MajesTEC-1 study [2]. The overall rate of CRS in our elderly cohort (52%) was lower than that reported in the MajesTEC-1 study (72.1%), and the ICANS rate was slightly higher (19% and 14.5%, respectively). Older patients often have lower organ reserves, including cardiac, pulmonary, and renal function [18], which may put them at higher risk for serious adverse events should CRS occur. However, it was encouraging to see that grade ≥3 events of CRS (*n* = 1) or ICANS (*n* = 3) were rare in the ≥75 age group. Importantly, these results were observed in a cohort of real-world RRMM patients, with most patients (73% in the older cohort) who would have been ineligible for MajesTEC-1.

A previous retrospective analysis by Dima et al., which was presented in abstract form, evaluated the outcomes of 33 patients aged >70 years old who received Tec in 5 US academic centers [9]. Overall patient characteristics were similar between the older and younger groups in that report, besides ECOG of ≥2, which was more prevalent in the >70 age group. This is in contrast to the current study, where we observed a lower incidence of adverse MM risk factors in the older age group, including HRCA and extramedullary disease, and a lower proportion of older patients in our cohort had received prior autologous stem cell transplantation or prior BCMA-directed therapy. Furthermore, in the current study, there was no difference in the performance status between the two age groups. The discrepancy between the two studies in baseline characteristics may be partially explained by the higher patient number in the current study, allowing for better elucidation of differences between the age groups. Nonetheless, similar to our study, the analysis by Dima et al. also did not observe significant differences between the two age groups in major efficacy and safety outcomes, including ORR (*p* = 0.37), median PFS (*p* = 0.61), CRS rates (*p* = 0.7) and ICANS rates (*p* = 0.17).

Our study has several inherent limitations, due to its retrospective and multicenter nature, including selection bias, variability in care, leading to inconsistencies in treatment and outcomes, and confounders that were unaccounted for despite the use of multivariable Cox regression analysis. Being a retrospective study, it is possible that several patients were inadvertently left out of this analysis, and data may have been incomplete and/or unavailable in some cases, affecting the reliability of the findings. However, each participating center was asked to submit all consecutive patients treated with teclistamab at their center for this analysis and other analyses for the U.S. Multiple Myeloma Immunotherapy Consortium. Moreover, a querying process to each center was utilized to address any inconsistent and/or missing data during the analysis. Due to unbalanced baseline patient and disease characteristics between the two age groups as described earlier, we were unable to make direct comparisons in survival outcomes. We also did not have access to frailty scores, which may be a more important determinant of outcomes in older patients than chronological age alone [19].

In conclusion, in this large, real-world multicenter study, we observed favorable safety and efficacy outcomes in elderly RRMM patients who received Tec. Rates of CRS and ICANS, as well as ORR and PFS, were comparable to those seen in the younger age group, as well as those seen in the pivotal MajesTEC-1 clinical trial. Our results suggest that Tec can be safely administered also in select patients aged ≥75 years old, and advanced age should not be the sole reason to preclude treatment. Instead, careful patient selection, based on functional status and comorbidities [20], should be employed to expand access to this important anti-myeloma modality.

## Supplementary information

Supplementary Table 1

Supplementary Table 2

Supplementary Table 3

## Data Availability

The underlying data of this study are available upon request to the corresponding author.

## References

[1] National Cancer Institute. Surveillance E, and End Results (SEER) Program. Cancer Stat Facts: Myeloma 2022. Available from: https://seer.cancer.gov/statfacts/html/mulmy.html.

[2] Moreau P, Garfall AL, van de Donk N, Nahi H, San-Miguel JF, Oriol A, et al. Teclistamab in relapsed or refractory multiple myeloma. N Engl J Med. 2022;387:495–505. 35661166 10.1056/NEJMoa2203478 PMC10587778

[3] Donk, NWCJvd, Moreau P, Garfall AL, Bhutani M, Oriol A, Nooka AK, et al. Long-term follow-up from MajesTEC-1 of teclistamab, a B-cell maturation antigen (BCMA) x CD3 bispecific antibody, in patients with relapsed/refractory multiple myeloma (RRMM). J Clin Oncol. 2023;41:8011

[4] Duma N, Azam T, Riaz IB, Gonzalez-Velez M, Ailawadhi S, Go R. Representation of minorities and elderly patients in multiple myeloma clinical trials. Oncologist. 2018;23:1076–8. 29700207 10.1634/theoncologist.2017-0592 PMC6192659

[5] Rajkumar SV. Multiple myeloma: 2024 update on diagnosis, risk-stratification, and management. Am J Hematol. 2024;99:1802–24. 38943315 10.1002/ajh.27422 PMC11404783

[6] Jones A, Bowcock S, Rachet B. Survival trends in elderly myeloma patients. Eur J Haematol. 2021;106:126–31. 33037667 10.1111/ejh.13530

[7] Martin TG, Mateos MV, Nooka A, Banerjee A, Kobos R, Pei L, et al. Detailed overview of incidence and management of cytokine release syndrome observed with teclistamab in the MajesTEC-1 study of patients with relapsed/refractory multiple myeloma. Cancer. 2023;129:2035–46. 36991547 10.1002/cncr.34756

[8] Dieterle MP, Mostufi-Zadeh-Haghighi G, Kus JW, Wippel C, Brugger Z, Miething C, et al. Safe and successful teclistamab treatment in very elderly multiple myeloma (MM) patients: a case report and experience from a total of three octogenarians. Ann Hematol. 2023;102:3639–41. 37710140 10.1007/s00277-023-05451-8 PMC10640491

[9] Dima D, Sannareddy A, Ahmed N, Davis JA, Shaikh H, Mahmoudjafari Z, et al. Toxicity and efficacy outcomes of teclistamab in patients with relapsed-refractory multiple myeloma (RRMM) above the age of 70 years: a multicenter study. Blood. 2023;142:3330.

[10] Harris PA, Taylor R, Thielke R, Payne J, Gonzalez N, Conde JG. Research electronic data capture (REDCap)-a metadata-driven methodology and workflow process for providing translational research informatics support. J Biomed Inf. 2009;42:377–81. 10.1016/j.jbi.2008.08.010 PMC2700030 18929686

[11] Zanwar S, Sidana S, Shune L, Puglianini OC, Pasvolsky O, Gonzalez R, et al. Impact of extramedullary multiple myeloma on outcomes with idecabtagene vicleucel. J Hematol Oncol. 2024;17:42. 38845015 10.1186/s13045-024-01555-4 PMC11157748

[12] Lee DW, Santomasso BD, Locke FL, Ghobadi A, Turtle CJ, Brudno JN, et al. ASTCT consensus grading for cytokine release syndrome and neurologic toxicity associated with immune effector cells. Biol Blood Marrow Transpl. 2019;25:625–38. 10.1016/j.bbmt.2018.12.758 PMC12180426 30592986

[13] Kumar S, Paiva B, Anderson KC, Durie B, Landgren O, Moreau P, et al. International Myeloma Working Group consensus criteria for response and minimal residual disease assessment in multiple myeloma. Lancet Oncol. 2016;17:e328–46. 27511158 10.1016/S1470-2045(16)30206-6

[14] Division UP. World population ageing 1950-2050 2002. Available from: chrome-extension://efaidnbmnnnibpcajpcglclefindmkaj/ https://www.un.org/development/desa/pd/sites/www.un.org.development.desa.pd/files/files/documents/2021/Nov/undesa_pd_2002_wpa_1950-2050_web.pdf.

[15] Weir HK, Thompson TD, Stewart SL, White MC. Cancer incidence projections in the United States between 2015 and 2050. Prev Chronic Dis. 2021;18:E59. 34114543 10.5888/pcd18.210006 PMC8220959

[16] Munshi PN, Vesole DH, St Martin A, Davila O, Kumar S, Qazilbash M, et al. Outcomes of upfront autologous hematopoietic cell transplantation in patients with multiple myeloma who are 75 years old or older. Cancer. 2021;127:4233–9. 34374445 10.1002/cncr.33831 PMC8699178

[17] Kalariya NM, Hildebrandt MAT, Hansen DK, Sidana S, Khouri J, Ferreri CJ, et al. Clinical outcomes after idecabtagene vicleucel in older patients with multiple myeloma: a multicenter real-world experience. Blood Adv. 2024;8:4679–88. 39042903 10.1182/bloodadvances.2024013540 PMC11402173

[18] Zhang H, Cherian R, Jin K. Systemic milieu and age-related deterioration. Geroscience. 2019;41:275–84. 31152364 10.1007/s11357-019-00075-1 PMC6702503

[19] Mian H, McCurdy A, Giri S, Grant S, Rochwerg B, Winks E, et al. The prevalence and outcomes of frail older adults in clinical trials in multiple myeloma: a systematic review. Blood Cancer J. 2023;13:6. 36599867 10.1038/s41408-022-00779-2 PMC9813365

[20] Palumbo A, Bringhen S, Mateos MV, Larocca A, Facon T, Kumar SK, et al. Geriatric assessment predicts survival and toxicities in elderly myeloma patients: an International Myeloma Working Group report. Blood. 2015;125:2068–74. 25628469 10.1182/blood-2014-12-615187 PMC4375104

