# Supplementary material for: Outcomes of elderly patients with relapsed refractory multiple myeloma (RRMM) treated with teclistamab: a multicenter study from the U.S. Multiple Myeloma Immunotherapy Consortium
Source: Blood Cancer J. 2025 May 9;15(1):92. doi: 10.1038/s41408-025-01297-7 (PMC12064690; doi:10.1038/s41408-025-01297-7)
Supplement: Supplementary file 1 — Supplementary Table 1 [file 41408_2025_1297_MOESM1_ESM.docx]

**Supplementary Table 1. Causes of death**

| **Measure** | **Age group** | |
| --- | --- | --- |
|  | **≥75 (Total=20)** | **<75 (Total=118)** |
| **Myeloma progression** | 14 (70%) | 89 (75%) |
| **Infection** | 5 (25%) | 12 (10%) |
| **Sepsis** | 2 (10%) | 6 (5%) |
| **Pneumonia** | 3 (15%) | 5 (4%) |
| **Unknown** | 0 | 1 (1%) |
| **Other malignancy** | 0 | 6 (5%) |
| **Other/unknown** | 1 (5%) | 11 (9%) |
