# Supplementary material for: Outcomes of elderly patients with relapsed refractory multiple myeloma (RRMM) treated with teclistamab: a multicenter study from the U.S. Multiple Myeloma Immunotherapy Consortium
Source: Blood Cancer J. 2025 May 9;15(1):92. doi: 10.1038/s41408-025-01297-7 (PMC12064690; doi:10.1038/s41408-025-01297-7)
Supplement: Supplementary file 2 — Supplementary Table 2 [file 41408_2025_1297_MOESM2_ESM.docx]

**Supplementary Table 2. Multivariable Analysis for PFS**

| **Parameter** | **Hazard Ratio (95% CI)** | **p-value** |
| --- | --- | --- |
| **Age** |  |  |
| <75 (vs. ≥75) | 1.15 (0.72 – 1.84) | 0.5538 |
| **Prior anti-BCMA agent** |  |  |
| Yes (vs. No) | 1.60 (1.13 – 2.28) | **0.0090** |
| **CRP upper quartile** |  |  |
| Yes (vs. No) | 1.61 (1.10 – 2.37) | **0.0146** |
| **Ferritin upper quartile** |  |  |
| Yes (vs. No) | 2.00 (1.32 – 3.01) | **0.0010** |
| **Hemoglobin <8 g/dL** |  |  |
| Yes (vs. No) | 1.90 (1.24 – 2.90) | **0.0032** |
| **Albumin <3 g/dL** |  |  |
| Yes (vs. No) | 1.41 (0.93 – 2.14) | 0.11 |
| **Plasma cell leukemia** |  |  |
| Yes (vs. No) | 4.93 (1.40 – 17.30) | **0.0128** |
| **Extramedullary disease** |  |  |
| Yes (vs. No) | 1.67 (1.17 – 2.38) | **0.0048** |

Variables considered from univariate analysis included: age, gender, race, high-risk cytogenetics, ultra high-risk myeloma, number of prior lines of therapy, triple/penta-refractoriness status, prior autologous transplant, prior anti-BCMA agent, prior anti-GPRC5D agent, CrCl<30, lactate dehydrogenase (LDH), Eastern Cooperative Oncology Group (ECOG), MajesTEC-1 eligibility, ferritin, C-reactive protein (CRP), platelets, Hemoglobin, absolute lymphocyte count (ALC), albumin, extramedullary disease (EMD), plasma cell leukemia (PCL), accelerated step-up dosing, bone marrow plasma cells
