# Supplementary material for: Outcomes of elderly patients with relapsed refractory multiple myeloma (RRMM) treated with teclistamab: a multicenter study from the U.S. Multiple Myeloma Immunotherapy Consortium
Source: Blood Cancer J. 2025 May 9;15(1):92. doi: 10.1038/s41408-025-01297-7 (PMC12064690; doi:10.1038/s41408-025-01297-7)
Supplement: Supplementary file 3 — Supplementary Table 3 [file 41408_2025_1297_MOESM3_ESM.docx]

**Supplementary Table 3. Multivariable Analysis for overall survival**

| **Parameter** | **Hazard Ratio (95% CI)** | **p-value** |
| --- | --- | --- |
| **Age** |  |  |
| <75 (vs. ≥75) | 1.67 (0.84 – 3.33) | 0.15 |
| **ECOG** |  |  |
| ≥2 (vs. <2) | 1.92 (1.17 – 3.16) | **0.0101** |
| **CRP upper quartile** |  |  |
| Yes (vs. No) | 2.58 (1.57 – 4.25) | **0.0002** |
| **Platelets <50 X10^9^/L** |  |  |
| Yes (vs. No) | 2.54 (1.50 – 4.23) | **0.0005** |
| **ALC <0.5 X10^9^/L** |  |  |
| Yes (vs. No) | 1.97 (1.21 – 3.21) | **0.0062** |
| **Plasma cell leukemia** |  |  |
| Yes (vs. No) | 5.24 (1.15, 23.84) | **0.0320** |

Variables considered from univariate analysis included: age, gender, race, high-risk cytogenetics, double-hit, number of prior lines of therapy, triple/penta-refractoriness status, prior autologous transplant, prior anti-BCMA agent, prior anti-GPRC5D agent, CrCl<30, LDH, ECOG, MajesTEC-1 eligibility, ferritin, CRP, platelets, Hemoglobin, ALC, albumin, EMD, PCL, accelerated step-up dosing, bone marrow plasma cells
